# ^177^Lu-Dotatate therapy for the treatment of metastatic neuroendocrine tumours in a patient on haemodialysis—dosimetric considerations

**DOI:** 10.1259/bjrcr.20150177

**Published:** 2015-08-18

**Authors:** Eleni Kalogianni, Danielle Louise Ruiz, Benjamin James Corcoran, Lindsey Ann Devlin, Gillian Claire Vivian, Nicola Jane Mulholland

**Affiliations:** Nuclear Medicine Department, King’s College Hospital Foundation Trust, London, UK

## Abstract

We report on a case of a 68-year-old female, currently a dialysis-dependant patient with disseminated metastatic neuroendocrine tumour, treated with ^177^Lu-Dotatate. As ^177^Lu-Dotatate is cleared predominantly by the kidneys, there are concerns regarding the treatment plan strategy to avoid increased radiation exposure compared with patients with normal renal function. For this purpose, personalized dosimetry was used to calculate the safe administered activity using whole-body scans. Employing this strategy allowed us to adjust the administered activity for the third fraction. The whole-body doses calculated were not significantly different from those received by patients with normal renal function. The radiological follow-up showed a stable disease, suggesting effective treatment. We found negligible radiation protection problems involved with this procedure.

Neuroendocrine tumours (NETs) are comparatively rare malignancies, which originate in the nervous or endocrine systems, characterized by their ability to produce hormones. Peptide receptor radionuclide therapy (PRRT) with somatostatin analogues labelled with β-emitting radionuclides has become one of the treatments of choice for treating NETs.

^177^Lu-Dotatate has gained popularity more recently as a PRRT agent. It has been demonstrated to be a safe, well-tolerated and effective treatment in patients with metastatic NET.^1,2^ Patients who are treated with ^177^Lu-Dotatate (^177^Lu-DOTA-D-Phe^1^-Tyr^3^) typically undergo four cycles each consisting of 7.4 GBq of the radiopharmaceutical at 6 to 10-week intervals.^3^

^177^Lu has a physical half-life of 6.73 days and emits β particles with a maximum energy of 149 keV and a maximum tissue penetration of 2 mm. In addition to the β particle emissions, ^177^Lu is also a two γ-photon emitter of low emission abundance at 113 keV (6.5%) and 208 keV (11%). Thus, quantitation of the γ-photons acquired using single-photon emission computed tomography (SPECT)/CT after the therapy can provide an estimate of the absorbed radiation dose.^4^

The amount of activity that can be administered in each cycle is constrained by the resultant dose to healthy organs; in this therapy regime, the bone marrow and kidneys are the dose-limiting organs.^5^ Renal impairment is a relative contraindication for PRRT therapy owing to its potential nephrotoxicity. However, this is not an issue in those with dialysis-dependent end-stage renal failure.

As ^177^Lu-Dotatate is cleared predominantly by the kidneys, patients undergoing chronic haemodialysis may potentially receive increased radiation exposure compared with patients with normal renal function. In this paper, we present a therapeutic procedure based on individualized dosimetry in order to minimize adverse events to the patient.

## Clinical presentation, diagnosis and imaging findings

**Figure 1.**
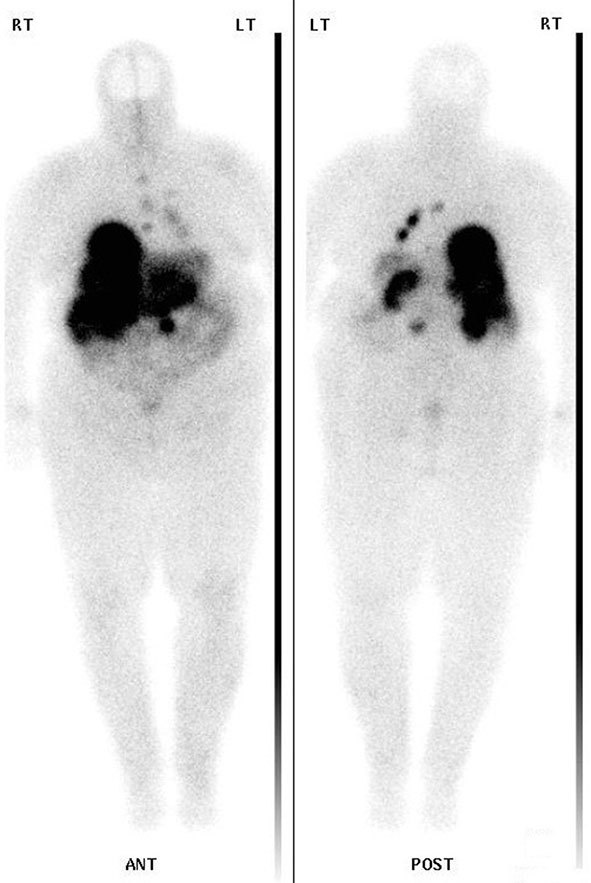
The 24-h whole-body image taken after the first ^177^Lu-Dotatate administration showing good therapeutic uptake in the liver and lung metastases.

The patient was a 68-year-old female with multiple endocrine neoplasia type 1. She had previous history of type 2 diabetes mellitus, parathyroidectomy, leiomyosarcoma and Factor V Leiden and was dialysis dependent. She had developed a grade 1 pancreatic NET 5 years earlier and was treated with distal pancreatectomy and splenectomy, followed by radiofrequency ablation to segment 6 liver metastases (Figure 1). She developed further liver metastases the following year, biopsy-proven MIB-1 30%, and was treated with eight cycles of carboplatin and etoposide. She remained symptomatic with lethargy, flushing and diarrhoea and was referred for PRRT for progressive liver and lung metastatic disease. This was proven somatostatin receptor positive on ^68^Ga-Dotatate positron emission tomography/CT imaging but ^123^I-metaiodobenzylguanidine (mIBG) negative.

## Treatment methods

The median percent of administered activity eliminated at 24 h from patients treated with^177^Lu-Dotatate has been shown to be 82.8%.^6^ In dialysis-dependent patients with no or negligible residual diuresis, ^177^Lu is not eliminated between consecutive haemodialysis sessions. As part of the treatment course, in order to restrict the radiation exposure, the first dialysis was performed within 24 h of therapy administration in all fractions.

The treatment plan consisted of three fractions of ^177^Lu-Dotatate therapy in a 15-month period. A clinical decision was made to reduce the administered activity to approximately 50% of the standard prescribed activity (7400 MBq) for the first two fractions to ensure that the whole-body radiation doses (WBDs) were within safe limits of the treatments.

Serial planar whole-body scans were acquired after the administration of ^177^Lu-Dotatate therapy on a hybrid dual-head SPECT-CT camera (Symbia T16 SPECT CT, Siemens Healthcare, Molecular Imaging, Hoffman Estates, IL), using medium-energy general-purpose collimators, peak at 208 keV (15% energy window, 512 × 1024 matrix size and 15 cm/min). The first scan was performed immediately after the administration of ^177^Lu-Dotatate so that the whole-body counts measured would determine the 100% of the injected activity. The subsequent scans were acquired at 24 h and on days 3–7 to coincide with the dialysis sessions.

Dosimetry was performed using the medical internal radiation dose (MIRD) scheme.^7^ The regions of interest over the anterior and the posterior whole-body scans were drawn manually using the HERMES hybrid viewer (Hermes Medical Solutions, Stockholm, Sweden). The geometric mean counts from the anterior and the posterior whole-body images were plotted over time, producing the time-dependent activities for whole-body regions.

**Figure 2.**
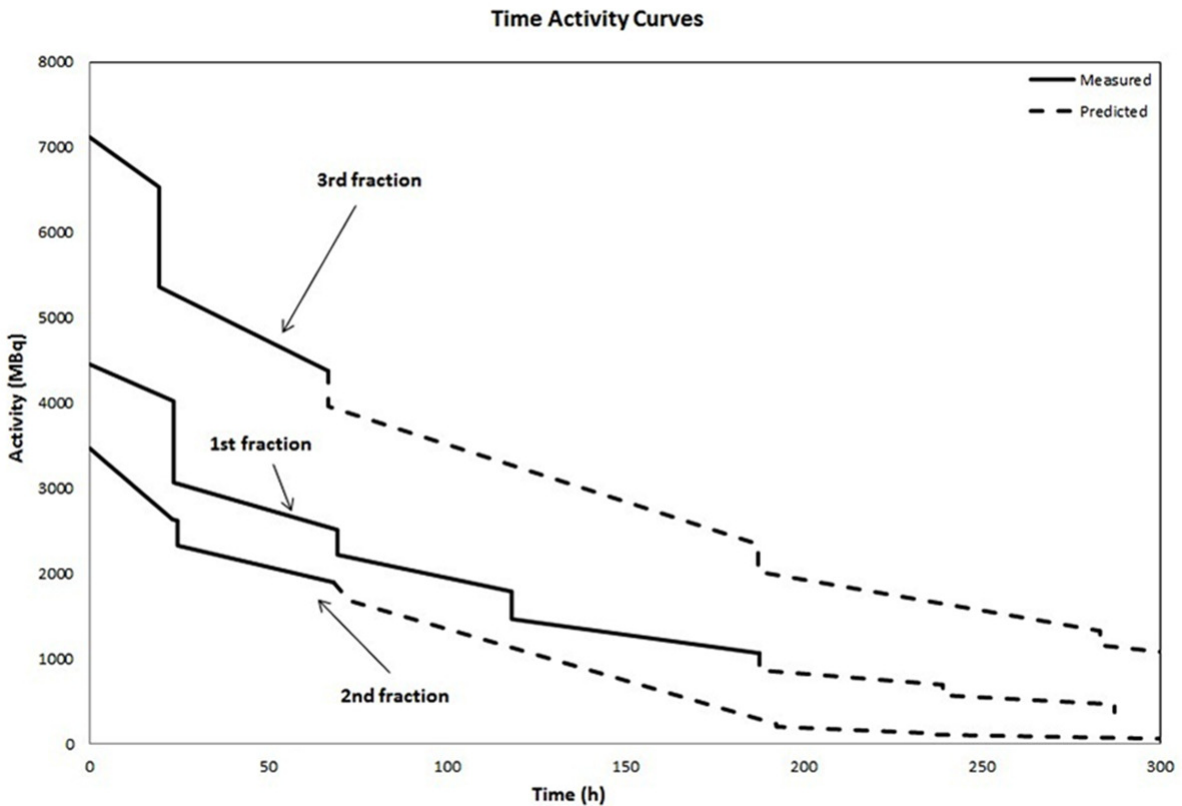
Time activity curves were generated from geometric mean of anterior and posterior counts from the whole-body scans for the three fractions of ^177^Lu-Dotatate administrations. Only physical decay of ^177^Lu was assumed to occur between two dialysis sessions. Because the loss of activity following dialysis was not constant over time, a mean decline in the fractional removal over time was calculated. The dashed line corresponds to the expected loss of ^177^Lu from patients.

Time activity curves (TACs) were generated for this patient by estimating the amount of ^177^Lu removed with each dialysis session, and assuming that only physical decay of ^177^Lu occurred between two dialysis sessions (Figure 2). The loss of activity following dialysis varied between sessions; therefore, a mean reduction in activity was calculated and used as an estimate of activity loss during subsequent dialysis. Then the WBDs were estimated by a simple multiplication of the cumulated activity, which was calculated using trapezoidal analysis from the TAC with the S-factor converting the ^177^Lu concentration into absorbed WBD (Equation 1):

(1) $$D_{WB}= ÃS_{WB}$$

Where:

- D_WB_ → (Gy) is the whole-body radiation dose.
- Ã → (MBq) is the cumulated activity.
- S_WB_ →(^m^Gyg/_M_Bqs) is the S-factor corrected for the weight of the patient.

Haemodialysis sessions were performed in a side room that was at a distance of at least 2 m from the nursing station. The usual procedures for biohazard protection with the safety rules for radiation protection were applied. The dialysis technicians and nurses wore electronic personal dosimeters (EPDs) to monitor radiation exposure when in contact with the patient during the haemodialysis sessions. At the completion of the dialysis session, the area and the equipment were monitored for ^177^Lu contamination.

## Results

During the first fraction, ^177^Lu removed was 23.7 and 11.8% for the first and second dialysis sessions, respectively. During the second fraction, ^177^Lu removed was 17.9 and 9.4% for the first and second dialysis sessions, respectively. The mean loss of activity following the dialysis was calculated as 18.0, 11.4 and 13.7% from the first, second and third sessions, respectively.

WBDs from the first two fractions were estimated as 0.6 and 0.5 Gy, respectively (administered activities 4450 and 3470 MBq, respectively), and were considered well tolerated with no immediate clinical complications and no change in the estimated glomerular filtration rate measurements on the pre-dialysis samples in the 6 weeks following each fraction.

Using the previous calculations, the estimated WBDs were determined assuming administration of the full prescribed activity of 7.4 GBq. The estimated WBDs were calculated as 0.9 and 0.7 Gy for the first and second treatments, respectively. These results were within the range of WBDs from patients treated with ^177^Lu-Dotatate not undergoing dialysis (0.1–1.1 Gy).^6^ As the first two treatments had been well tolerated clinically and dosimetry was repeatedly within a safe range, it was decided to administer the standard activity (7400 MBq). Using the previous methodology, the WBD from the third fraction was calculated as 0.7 Gy (administered activity 7117 MBq).

A previous risk assessment had shown negligible radiation hazard to the ward staff involved in the patient's management, with no contamination of the dialysis equipment. The patient was dialyzed in an isolated room for the first two sessions and the radiation safety personnel monitored all involved personnel and equipment. The radioactivity excreted by the patient during dialysis was discharged through the drains via the dialysis unit drain tube. Disposable lines and filters from the dialysis room were bagged and stored as radioactive waste. The dialysis nurses were advised to stay at a minimum distance of 1 m from the patient when possible. The EPDs verified that the nurses did not receive any measureable exposure.

## Outcome, follow-up and discussion

The patient had symptomatic improvement with reduced lethargy and diarrhoea, although the latter was not completely controlled, over the 18-month period of her ^177^Lu therapies. There was no grade 1 or above World Health Organization toxicity reported following the treatment. The patient died 2 years post ^177^Lu therapy following a chest infection, with an overall survival of 7 years from the time of diagnosis.

This case study shows that the fraction of ^177^Lu removed following dialysis decreases as more of the Dotatate becomes bound to the receptors. The same effect has been described in the literature when radioiodine treatments were administered to patients undergoing haemodialysis.^8^ Although ^177^Lu-Dotatate is not suitable in patients with renal impairment owing to nephrotoxicity, it appears safe for use in patients with end-stage renal failure on renal haemodialysis.

## Learning points

Based on the experience from this case, we can conclude that:

1. ^177^Lu-Dotatate therapy may be performed safely in patients on renal dialysis.
2. Dosimetry can be used to plan the therapy to ensure that WBDs are within safe limits.
3. For radiation protection purposes, at least the first two sessions of dialysis should be performed in an isolated room and the dialysis waste should be stored for decay.
